# Ultrasonic irradiation assisted efficient regioselective synthesis of CF_3_-containing pyrazoles catalyzed by Cu(OTf)_2_/Et_3_N

**DOI:** 10.1186/1752-153X-7-101

**Published:** 2013-06-13

**Authors:** Abdullah S Al-Bogami, Tamer S Saleh, Hassan M Albishri

**Affiliations:** 1 Chemistry Department, Faculty of Science, King Abdulaziz University, North Jeddah, P.O box 80203, Jeddah 21589, Saudi Arabia; 2 Green Chemistry Department, National Research Centre, Dokki, Cairo 12622, Egypt

**Keywords:** Ultrasonic irradiation, Pyrazole, Trifluromethyl moiety, HMBC, Copper(II)triflate

## Abstract

**Background:**

Most of the known approaches to the synthesis of CF_3_-containing organic compounds suffer from serious drawbacks. For example the starting materials required for these methods are rather difficult to obtain, or they are fairly toxic and inconvenient to work with and methods for direct fluorination and trifluoromethylation do not always allow the introduction of the CF_3_-group at the required position of a molecule.

**Results:**

An efficient and attractive regioselective synthesis of a series of novel pyrazoles containing the trifluromethyl moiety was achieved using Cu(OTf)_2_/Et_3_N as an efficient catalytic system under ultrasonic irradiation.

**Conclusions:**

Cu(OTf)_2_/Et_3_N catalyst showed a great advantage over all the investigated catalysts, and the ultrasonic irradiation method offered high yields of pyrazoles in short reaction time compared with classical conditions. gHMBC spectra of the product were used to rationalize the observed regioselectivity.

## Background

**Figure 1:**
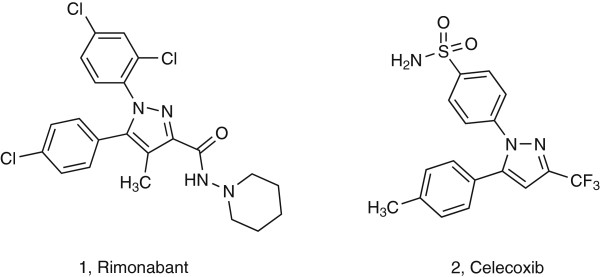
Examples of pharmaceutically relevant pyrazoles.

Over the last two decades there has been rapid progress in synthetic organic chemistry associated with the search for new organic compound derivatives with desirable properties. Such compounds are widely used in the pharmaceutical industry. Among the several FDA approved pharmaceutical drugs, the pyrazole core is found in rimonabant **(1)**, and celecoxib **(2)** (Figure 1) [1].

Pyrazoles exhibit significant biological properties such as antihyperglycemic [2], analgesic [3], anti-inflammatory [4], antipyretic [5], antibacterial [6], hypoglycemic [7] and sedative-hypnotic activities [8].

The trifluoromethyl group is a very important substituent in medicinal chemistry, due to its unique stereoelectronic properties. A trifluoromethyl group usually increases the lipophilicity of a molecule, improving its transport characteristics in vivo. Furthermore, the strength and durability of the C-F bond compared with the С-Н bond (116 and 100 kcal/mol respectively) allows undesirable metabolic transformations to be avoided. So the introduction of trifluoromethyl groups into bioactive molecules has become very important in pharmaceutical studies, stimulating work directed towards the elaboration of synthetic methodology for compounds containing trifluoromethyl groups. Because of all these factors, organofluorine chemistry has been vigorously developing during the past two decades [9].

Most of the known approaches to the synthesis of CF_3_-containing organic compounds suffer from serious drawbacks. First of all, the starting materials required for these methods are rather difficult to obtain, or they are fairly toxic and inconvenient to work with. Additionally, methods for direct fluorination and trifluoromethylation do not always allow the introduction of the CF_3_-group at the required position of a molecule.

It is worth mentioning that most of the reported methods for synthesis of CF_3_-containing pyrazoles suffer from formation of mixures of regioisomers. Pavlik *et al.* studied the reactivity of CF_3_-enones towards *N*-substituted hydrazines where a mixture of regioisomers was obtained. Also, in the reaction of *β*-alkoxy substituted enones with *N*-methyl hydrazine two isomeric dihydropyrazoles were obtained [10,11]. Singh *et al.* investigated the reactivity of 3-(ethoxymethylene)1,1,1-trifluropentan-2,4-dione with various aryl and heteroaryle substituted hydrazines, and a mixure of pyrazoles formed [12].

Nenajdenko *et al.* obtained CF_3_-conatining pyrazoles with 100% regioselectivty from the reaction of α-bromo-ethoxy-trifluromethyl enone with aryl hydrazine, but with lower yield [13].

As a result the more flexible “synthon” approach, based on the application of simple and readily available fluorine-containing compounds has gained substantial interest [14]. Nitrilimines containing a fluro or trifluoromethyl moiety (liberated in situ from the corresponding hydrazonyl halide) are easily available compounds which can be prepared by various methods [15] and fairly convenient building blocks to prepare heterocyclic compounds containing a trifluoromethyl group.

It is evident from the recent literature that copper triflate [Cu(OTf)_2_] has invoked enormous interest as a green and potential Lewis acid catalyst to construct carbon–carbon and carbon– heteroatom bonds in various organic transformations [16-23]. Despite its great importance, only a few papers have reported on its catalytic application in organic synthesis [24].

Ultrasound irradiation has been utilized to accelerate a number of synthetically useful reactions during the last few years. Cavitation is the formation, growth and collapse of bubbles in an irradiated liquid. This effect induces very high local pressure and temperatures inside the bubbles and enhances mass transfer and turbulent flow in the liquid [25]. Ultrasound has been utilized to accelerate a number of synthetically useful reactions, especially in heterocyclic chemistry [26-36].

As part of our ongoing interest in sonochemistry [37-42], and in a continuation of our interest in the synthesis of a wide range of heterocyclic systems for biological screening programme in our laboratory [43-51], we will introduce here a novel and efficient regioselective synthesis of trifluromethyl containing pyrazoles under ultrasonic irradaition promoted by catalytic amount of copper triflate and triethylamine.

## Results and discussion

**Scheme 1:**
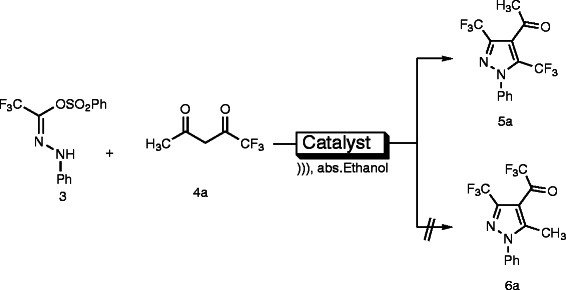
Regioselective synthesis of pyrazole derivative 5a using various catalysts under ultrasonic irradiation. Regioselective synthesis of pyrazole derivative 5a using various catalysts under ultrasonic irradiation.

A wide variety of catalysts were scanned for the reaction of *N*-phenyltrifluromethylcarbohydrazonoyl benzenesulfonate **(3)** and 1,1,1-trifluoropentane-2,4-dione **(4a)** (Scheme 1) in ethanol under ultrasound irradiation as a model reaction (Table 1). These catalysts are sodium ethoxide, triethylamine, copper triflate, copper triflate/triethyl amine, which were selected to promote the mentioned reaction.

**Table 1:** The yield of pyrazole derivative 5a obtained using various catalysts under ultrasonic irradiation

**Entry**	**Catalyst**	**Loading (mol%)**	**Time (min.)**	**Yields (%)**
1	None	-	180	No reaction
2	EtONa	100	60	Traces
3	Et_3_N	5	45	73
4	Et_3_N	10	45	67
5	Cu(OTf)_2_	5	60	No reaction
6	Cu(OTf)_2_	10	60	No reaction
7	Cu(OTf)_2_/ Et_3_N	5:5	45	90
8	Cu(OTf)_2_/ Et_3_N	10:5	60	90

Absolute ethanol was selected as solvent due to the solubility of the two starting materials. Thin layer chromatography (TLC) used to follow the reaction progress.

It is clear from the results cited in Table 1, that only one isolable unique product was obtained (as examined by TLC). The best yield of 90% of the isolated product was reached using Cu(OTf)_2_ and Et_3_N, each of 0.05 equiv. (entry 7). The reaction gave relatively low yield when 0.05 or 0.1 equiv. of Et_3_N alone was used (entry 3,4). Furthermore, sodium ethoxide base gave a trace amount of product (entry 2) and no reaction was observed in the absence of catalyst or using Cu(OTf)_2_ as catalyst in 5 or 10 mol% (entry 1,5,6 respectively). Also from Table 1, the effect of loading (Mol%) on the% yield of isolated product was studied, and it is clear that 0.05:0.05 equiv. of Cu(OTf)_2_ and Et_3_N furnishes the respective product in a quantitative yield (Table 1, entry 7,8).

It is worth mentioning that we followed the above reaction to completion using GC-MS and the resulting products were identified from their retention times. It was found that 100% regioselectivity was attained in presence of Cu(OTf)_2_ with Et_3_N (Table 1, entry 7 and 8) but in the presence Et_3_N only (Table 1, entry 3 and 4) there was a very small amount (traces) of other product detected by GC-MS, which completely disappeared in recrystalization of the crude product to give only pure **5a**.

**Figure 2:**
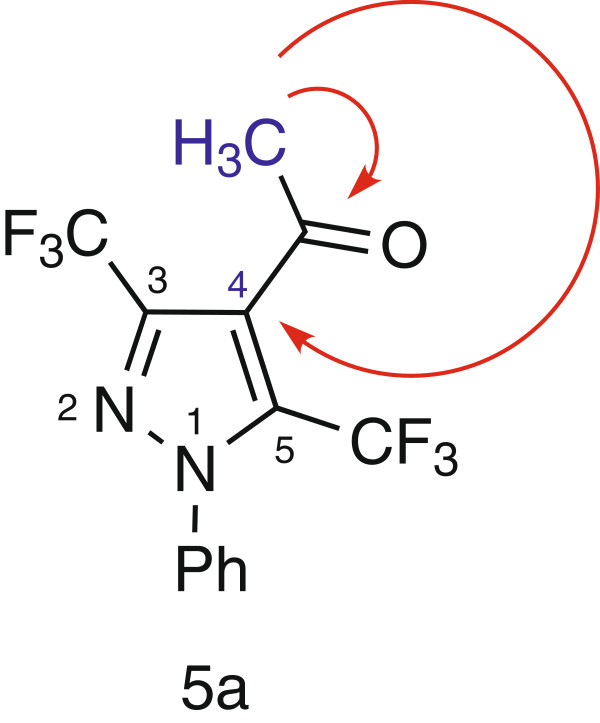
Diagnostic correlations in the gHMBC (red arrows) in compound 5a.

The isolated product was identified as the pyrazole structure **5a**, although two regioisomeric cycloadducts **5a** and **6a** seemed possible (Scheme 1). Unambiguous structure determination of the obtained products is therefore crucial to rationalize the observed regioselectivity. Structure elucidation was conveniently achieved on the basis of the ^1^H-^13^C connectivities (gHMBC) showed by methyl protons towards the carbonyl group and C-4 of pyrazole. On this basis, the analysis of gHMBC spectra of the product led us to attribute the signal at δ 191.65 ppm to the quaternary carbon (carbonyl group) owing to its correlation peaks with the methyl protons at δ 2.65 ppm; also, the signal at δ 112.11 ppm to the C-4 of the pyrazole owing to its correlation peaks with the methyl protons at δ 2.65 ppm. This observation indicated that the methyl protons correlate to carbonyl functional group and the C-4 of the pyrazole, which is in accordance to the structure **5a** (Figure 2). Therefore, ^1^H-^13^C HMBC of the product formed provides sharp evidence for the existence of regioisomer **5a** and rules out the other alternative structure **6a**. Consequently the reaction product was identified as the pyrazole structure **5a** (Scheme 1).

**Figure 3:**
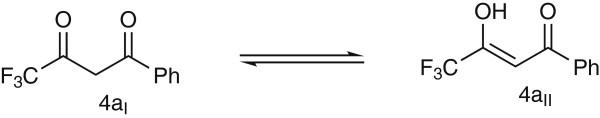
The carbonyl group adjacent to the perfluoroalkyl chain enolizes preferentially.

The formation of pyrazole product **5a** is in line with the well established earlier studies for *β*-diketones containing highly fluorinated groups, in which the enolic content predominates, as in Figure 3[52-54].

**Scheme 2:**
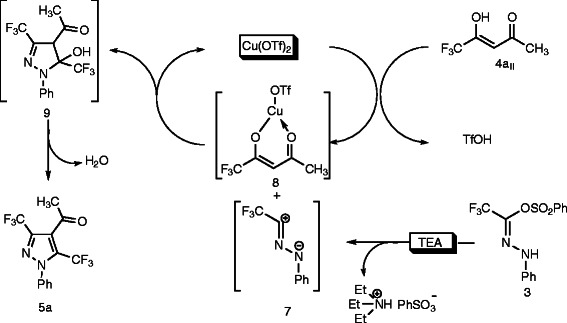
A Proposed Mechanism for Cu(OTf)_2_/TEA-Catalyzed regioselective synthesis of pyrazole. **A Proposed Mechanism for Cu(OTf)**_**2**_**/TEA-Catalyzed regioselective synthesis of pyrazole.**

It is reasonable to propose a mechanism for formation of the pyrazole **5a** under the adopted reaction conditions (Scheme 2). Pyrazole **5a** was assumed to form *via* nitrile imine **7** (liberated *in situ* from **3** by the action of triethylamine and release triethyl ammonium salt) which involved a more or less concerted cycloaddition reaction. In the Cu(OTf)_2_-catalyzed addition reactions it reacts with the enolized form of *β*-diketone **4a**_**II**_ to generate copper(II) intermediate **8** and release of triflic acid (TfOH). The nitilie imine **7** reacts with intermediate **8** to give non isolable intermediate 5-hydroxy-2-pyrazoline derivative **9**, and cyclizes *via* elimination of molecule of water to yield the desired regioselective pyrazole derivatives **5a**.

Therefore, copper(II)triflate has an important role in which it activates the enolic O-H bond of *β*-diketone to initiate the reaction.

**Scheme 3:**
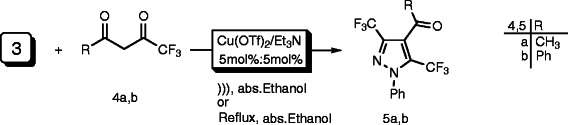
Regioselective synthesis of pyrazole derivatives 5a,b using Cu(OTf)_2_/Et_3_N catalyst under both ultrasonic irradiation and conventional method. **Regioselective synthesis of pyrazole derivatives 5a,b using Cu(OTf)**_**2**_**/Et**_**3**_**N catalyst under both ultrasonic irradiation and conventional method.**

The scope and generality of this protocol was tested using another derivative of *β*-diketone **4b** with *N*-phenyltrifluromethylcarbo-hydrazonoyl benzenesulfonate **(3)** as shown in the Scheme 3 under the optimized reaction conditions, and the corresponding pyrazole derivatives **5b** were obtained in excellent yield.

Also, to find the specific effect of ultrasound on these reactions, all previously mentioned reactions were carried out under the same conditions in the absence of ultrasound irradiation (Table 2). It was observed that the reaction time increased considerably and the yield of the products decreased. Thus, ultrasound was found to have a beneficial effect on the synthesis of pyrazole derivatives, in which the time of the above reactions decreased from 8 to 10 h in the conventional procedure to less than 1 h. Also, there was an improvement in the yields of the reactions under ultrasonic irradiations.

**Table 2:** **The yields of pyrazole derivatives 5a,b using Cu(OTf)**_**2**_**/Et**_**3**_**N catalyst under conventional and ultrasonic irradiations conditions**

**Entry**	**Product**	**Ultrasonic irradiation**		**Silent condition**	
		**Time (min.)**	**Yield%**	**Time (h)**	**Yield%**
**1**	**5a**	**45**	**90**	**6**	**80**
**2**	**5b**	**45**	**88**	**8**	**78**

**Scheme 4:**
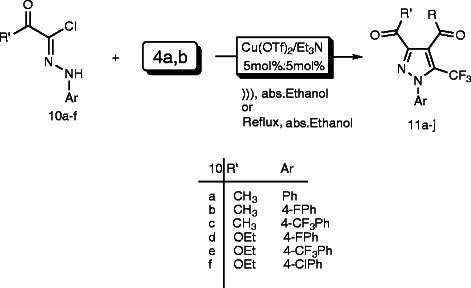
Regioselective synthesis of pyrazole derivatives 11a-l using u(OTf)_2_/Et_3_N catalyst under both ultrasonic irradiation and conventional method. **Regioselective synthesis of pyrazole derivatives 11a-l using u(OTf)**_**2**_**/Et**_**3**_**N catalyst under both ultrasonic irradiation and conventional method.**

We extended our study to find out the reactivity of *β*-diketone **4a,b** towards the *α*-ketohydrazonoyl halides **10a-f** under ultrasonic irradiation and/or conventional method to give the corresponding pyrazole derivatives **11a-l** in 85–91% yields (Scheme 4, Table 3).

**Table 3:** Synthesis of pyrazole 11a–l under both ultrasonic irradiation and conventional method

**Compound no.**	**Product**	**Ultrasonic irradiation**		**Silent condition**	
		**Time (min.)**	**Yield%**	**Time (h)**	**Yield%**
**11a**		**45**	**90**	**7**	**80**
**11b**		**45**	**88**	**7**	**76**
**11c**		**45**	**90**	**6**	**77**
**11d**		**45**	**88**	**8**	**75**
**11e**		**45**	**88**	**10**	**76**
**11f**		**45**	**85**	**10**	**76**
**11g**		**60**	**89**	**10**	**76**
**11h**		**60**	**90**	**10**	**77**
**11i**		**60**	**85**	**11**	**73**
**11j**		**60**	**85**	**11**	**77**
**11k**		**60**	**86**	**9**	**75**
**11l**		**60**	**90**	**10**	**77**

The structure of pyrazole derivatives **11a–l** was assigned on the basis of their elemental analyses and spectral data. For example, the ^1^H NMR spectrum of compound **11a** revealed two singlet signals at δ 2.29 and 2.74 due to the two methyl groups in addition to aromatic multiplet at δ 7.31–7.71. The mass spectrum of the same compound revealed a peak corresponding to its molecular ion at *m/z* 296. The mechanism of the formation of compounds **11a-l** is in line with those depicted in Scheme 2.

From the data cited in Table 3, it was observed that the reaction time increased considerably and the yield of the products decreased under conventional method. Thus, ultrasound was found to have beneficial effect on the synthesis of pyrazole derivatives in which decrease time of above reactions from 7 to 11 h in conventional procedure to 45 min. to 1 h. Also, there was a noticeable improvement in the yields of the reactions under ultrasonic irradiations.

The improvement induced by ultrasound in the above mentioned reactions can be attributed to the well established theory for the cavitation, The collapse of bubbles caused by cavitation produces intense local heating and high pressures [55,56], so reaction time decreases clearly and high% yield was obtained.

## Conclusion

An ultrasonic assisted efficient protocol for the regioselective synthesis a series of novel pyrazoles containing the trifluromthyl moiety utilizing an efficient catalytic system Cu(OTf)_2_/Et_3_N was reported. Cu(OTf)_2_/Et_3_N catalyst showed a great advantage over all the investigated catalysts and ultrasonic irradiation method offered high yields of pyrazoles in short reaction times compared with classical conditions. gHMBC spectra of the product were used to rationalize the observed regioselectivity.

## Experimental

### General

All organic solvents were purchased from commercial sources and used as received unless otherwise stated. All chemicals were purchased from Merck, Aldrich or Acros and used without further purification, thin-layer chromatography (TLC) was performed on precoated Merck 60 GF254 silica gel plates with a fluorescent indicator, and detection by means of UV light at 254 and 360 nm. All melting points were measured on a Stuart melting point apparatus and are uncorrected. IR spectra were recorded in IR spectra were recorded in the Smart iTR which is an ultra-high-performance, versatile Attenuated Total Reflectance (ATR) sampling accessory on The Nicolet iS10 FT-IR spectrometer. The NMR spectra were recorded on a Varian Mercury VX-300 NMR spectrometer. ^1^H spectra were run at 300 MHz and ^13^C spectra were run at 75.46 MHz in dimethyl sulphoxide (DMSO-d_6_). Chemical shifts were related to that of the solvent. Assignments are made using ^1^H, ^13^C, gHMBC, 2D experiments was done using standard Varian methods.

Mass spectra were recorded on the Thermo ISQ Single Quadrupole GC-MS. The resulting products were identified from their retention times by GC–MS analysis. Elemental analyses were carried out on Euro*V*ector instrument C, H, N, S analyzer EA3000 Series. Sonication was performed by Techno-gaz sonicator (with a frequency of 37 kHz and ultrasonic peak max. 320 W).

*N*-phenyltrifluromethylcarbohydrazonoyl benzenesulfonate **(3)**[15], *α*-ketohydrazonoyl halides **10a-f**[57] were prepared according to the reported literature.

### General procedure and characterization data

Typical procedure for synthesis of pyrazole derivatives 5a,b and 11a-j.

### Sonicated reactions

In an Erlenmeyer flask, a mixture of *β*-diketone **(4a,b)** (1 mmol) and appropriate hydrazonoyl benzenesulfonate **(3)** and/or hydrazonyl halides **(10a-e)** (1 mmol) **(3)** was taken in absolute ethanol (30 ml) in the presence of Cu(OTf)_2_/Et_3_N (5:5 mol%) as catalyst then subjected to ultrasonic irradiations for appropriate time (*cf.* Tables 1, 2, 3). All The reactions were kept at 70–80°C (the temperature inside reaction vessel was 70–76°C and the reaction flask was put in the mid of sonicator bath to to achieve effective cavitations). The sonochemical reactions were continued until the starting materials were no longer detectable by TLC. After the completion of the reaction, EtOAc (30 mL) was added to dilute the reaction solution. Then, the mixture was washed with water. The combined organic phases were dried and concentrated in *vacuo*, and the remaining residue was purified by recrystalization from ethanol to afford pyrazole derivatives **5a,b** and **11a-j.** The above reaction was studied also by using various condition and catalysts.

(i) in absence of catalyst: this process was performed as described above and no product formed (ii) in presence of equivalent amount from sodium methoxide, this process was performed as described above and only a trace of product obtained identified as **5a** (*cf.* Table 1) (iii) in presence of different ratio of triethylamine (0.05 mmol) or (0.1 mmol) also, these processes were performed as described above and the progress of the reaction was monitored by TLC, the product formed was identified as **5a** in each case (*cf.* Table 1) in different percent yield, (iv) in presence of different ratio of copper (II) triflate (0.05 mmol) or (0.1 mmol) also, these processes were performed as described above and no product formed.

### Silent reactions

These processes were performed on the same scale described above for sonicated reaction. Here the reactant and catalyst were put in ethanol under reflux for suitable time (*cf.* Tables 2 and 3) until the starting materials were no longer detectable by TLC. The products were obtained and purified as described above in sonicated reaction.

### Physical and spectral data of the compounds 5a,b and 11a-j are listed below

#### 4.2.2.1. 4-Acetyl-3,5-di(trifluoromethyl)-1-phenylpyrazole (5a) [58]

M.p. = 211–213°C; IR (KBr): 1712 (C = O), 1611 (C = N) cm^-1^; ^1^H NMR (300 MHz, CDCl_3_) δ: 2.65 (s, 3H, CH_3_), 6.52-7.23 (m, 5H, ArH’s); ^13^C NMR (75.46 MHz, CDCl_3_) δ: 31.42, 112.11, 118.00, 119.58, 123.89, 126.24, 129.30, 136.87, 139.00, 154.62, 191.65. MS (m/z): 322 (M^+^). (Calc.: C, 48.46; H, 2.50; N, 8.69. C_13_H_8_F_6_N_2_O Found: C, 48.72; H, 2.38; N,8.55).

#### 4-Benzoyl-3,5-di(trifluoromethyl)-1-phenylpyrazole (5b)

M.p. = 227–228°C; IR (KBr): 1708 (C = O), 1601 (C = N) cm^-1^; ^1^H NMR (300 MHz, CDCl_3_) δ: 7.22-7.69 (m, 10H, ArH’s); ^13^C NMR (75.46 MHz, CDCl_3_) δ: 116.05, 119.18, 121.94, 125.54, 126.33, 128.52, 128.53, 129.14, 130.25, 136.89, 139.34, 139.74, 152.87, 193.16. MS (m/z): 384 (M^+^). (Calc.: C, 56.26; H, 2.62; N, 7.29. C_18_H_10_F_6_N_2_O Found: C, 56.50; H, 2.52; N,7.15).

#### 3,4-Diacetyl-1-phenyl-5-trifluoromethylpyrazole (11a)

M.p. = 192–194°C; IR (KBr): 1712, 1699 (2 C = O), 1594 (C = N) cm^-1^; ^1^H NMR (300 MHz, DMSO-d_6_) δ: 2.29 (s, 3H, CH_3_), 2.74 (s, 3H, CH_3_), 7.31-7.71 (m, 5H, ArH’s); ^13^C NMR (75.46 MHz, DMSO-d_6_) δ: 29.15, 31.54, 115.15, 122.01, 125.53, 125.54, 127.41, 129.14, 131.06, 139.98, 150.98, 196.24, 199.09. MS (m/z): 296 (M^+^). (Calc.: C, 56.76; H, 3.74; N, 9.46. C_14_H_11_F_3_N_2_O_2_ Found: C, 56.50; H, 2.52; N,7.15).

#### 3-Acetyl-4-benzoyl-1-phenyl-5-trifluoromethylpyrazole (11b)

M.p. = 213–214°C; IR (KBr): 1710, 1701 (2 C = O), 1599 (C = N) cm^-1^; ^1^H NMR (300 MHz, DMSO-d_6_) δ: 2.24 (s, 3H, CH_3_), 7.16-7.67 (m, 10H, ArH’s); ^13^C NMR (75.46 MHz, DMSO-d_6_) δ: 28.98, 115.24, 120.07, 124.13, 126.22, 128.11, 128.12, 129.85, 133.54, 139.47, 152.00, 194.01, 198.19. MS (m/z): 358 (M^+^). (Calc.: C, 63.69; H, 3.66; N, 7.82. C_19_H_13_F_3_N_2_O_2_ Found: C, 63.96; H, 3.14; N,7.67).

#### 3,4-Diacetyl-1-(4-flurophenyl)-5-trifluoromethylpyrazole (11c)

M.p. = 175–177°C; IR (KBr): 1711, 1697 (2 C = O), 1601 (C = N) cm^-1^; ^1^H NMR (300 MHz, DMSO-d_6_) δ: 2.31 (s, 3H, CH_3_), 2.59 (s, 3H, CH_3_), 7.24 (d, 2H, *J =* 8.4 Hz, ArH’s), 7.55 (d, 2H, *J =* 8.4 Hz, ArH’s); ^13^C NMR (75.46 MHz, DMSO-d_6_) δ: 26.94, 31.20, 113.54, 115.23, 119.08, 123.94, 132.15, 137.65, 152.89, 162.41, 195.00, 198.23. MS (m/z): 314 (M^+^). (Calc.: C, 53.51; H, 3.21; N, 8.91. C_14_H_10_F_4_N_2_O_2_ Found: C, 53.78; H, 3.10; N,8.75).

#### 3-Acetyl-4-benzoyl-1-(4-flurophenyl)-5-trifluoromethylpyrazole (11d)

M.p. = 201–203°C; IR (KBr): 1719, 1701 (2 C = O), 1603 (C = N) cm^-1^; ^1^H NMR (300 MHz, DMSO-d_6_) δ: 2.28 (s, 3H, CH_3_), 7.21-7.84 (m, 9H, ArH’s); ^13^C NMR (75.46 MHz, DMSO-d_6_) δ: 27.98, 112.03, 115.87, 119.26, 125.44, 128.31, 132.05, 135.16, 136.49, 149.97, 162.00, 191.24, 196.20. MS (m/z): 376 (M^+^). (Calc.: C, 60.64; H, 3.21; N, 7.44. C_19_H_12_F_4_N_2_O_2_ Found: C, 60.95; H, 3.07; N,7.27).

#### 3,4-Diacetyl-1-(4-trifluoromethylphenyl)-5-trifluoromethylpyrazole (11e)

M.p. = 194–196°C; IR (KBr): 1707, 1697 (2 C = O), 1600 (C = N) cm^-1^; ^1^H NMR (300 MHz, DMSO-d_6_) δ: 2.26 (s, 3H, CH_3_), 2.61 (s, 3H, CH_3_), 7.44 (d, 2H, *J =* 8.1 Hz, ArH’s), 7.67 (d, 2H, *J =* 8.1 Hz, ArH’s); ^13^C NMR (75.46 MHz, DMSO-d_6_) δ: 29.91, 32.42, 117.21, 123.94, 124.36, 124.85, 126.03, 129.54, 138.03, 143.11, 151.98, 192.10, 196.85. MS (m/z): 364 (M^+^). (Calc.: C, 49.46; H, 2.77; N, 7.69. C_15_H_10_F_6_N_2_O_2_ Found: C, 49.75; H, 2.63; N,7.54).

#### 3-Acetyl-4-benzoyl-1-(4-trifluoromethylphenyl)-5-trifluoromethyl- pyrazole (11f)

M.p. = 210°C; IR (KBr): 1706, 1699 (2 C = O), 1598 (C = N) cm^-1^; ^1^H NMR (300 MHz, DMSO-d_6_) δ: 2.26 (s, 3H, CH_3_), 7.42-7.85 (m, 9H, ArH’s); ^13^C NMR (75.46 MHz, DMSO-d_6_) δ: 27.58, 113.13, 119.26, 121.35, 124.08, 125.65, 127.11, 128.45, 129.69, 132.45, 134.00, 135.04, 143.01, 149.19, 190.98, 196.01. MS (m/z): 426 (M^+^). (Calc.: C, 56.35; H, 2.84; N, 6.57. C_20_H_12_F_6_N_2_O_2_ Found: C, 56.68; H, 2.71; N,6.37).

#### Ethyl 4-acetyl-1-(4-fluorophenyl)-5-(trifluoromethyl)-1H-pyrazole-3-carboxylate (11 g)

M.p. = 180–182°C; IR (KBr): 1723, 1699 (2 C = O), 1598 (C = N) cm^-1^; ^1^H NMR (300 MHz, DMSO-d_6_) δ: 1.29 (t, 3H, *J =* 7.2 Hz, CH_3_), 2.23 (s, 3H, CH_3_), 4.01(q, 2H, *J =* 7.2 Hz, CH_2_), 7.28 (d, 2H, *J =* 8.4 Hz, ArH’s), 7.65 (d, 2H, *J =* 8.4 Hz, ArH’s); ^13^C NMR (75.46 MHz, DMSO-d_6_) δ: 13.11, 29.18, 58.11, 113.26, 119.01, 125.36, 132.58, 136.00, 148.39, 160.07, 162.37, 192.10. MS (m/z): 344 (M^+^). (Calc.: C, 52.33; H, 3.51; N, 8.14. C_15_H_12_F_4_N_2_O_3_ Found: C, 52.61; H, 3.38; N,7.99).

#### Ethyl 4-benzoyl-1-(4-fluorophenyl)-5-(trifluoromethyl)-1H-pyrazole-3-carboxylate (11h)

M.p. = 212°C; IR (KBr): 1725, 1701 (2 C = O), 1598 (C = N) cm^-1^; ^1^H NMR (300 MHz, DMSO-d_6_) δ: 0.91 (t, 3H, *J =* 7.6 Hz CH_3_), 4.23 (q, 2H, *J =* 7.6 Hz, CH_2_), 7.21-7.78 (m, 9H, ArH’s); ^13^C NMR (75.46 MHz, DMSO-d_6_) δ: 13.26, 58.25, 112.31, 115.25, 118.01, 119.27, 125.87, 128.97, 131.98, 132.68, 134.85, 137.50, 148.05, 159.98, 163.00, 194.28. MS (m/z): 406 (M^+^). (Calc.: C, 59.12; H, 3.47; N, 6.89. C_20_H_14_F_4_N_2_O_3_Found: C, 59.39; H, 3.34; N,6.75).

#### Ethyl 4-acetyl-1-(4-trifluoromethylphenyl)-5-(trifluoromethyl)-1H-pyrazole-3-carboxylate (11i)

M.p. = 192–194°C; IR (KBr): 1721, 1699 (2 C = O), 1599 (C = N) cm^-1^; ^1^H NMR (300 MHz, DMSO-d_6_) δ: 0.89 (t, 3H, *J =* 7.2 Hz, CH_3_), 2.29 (s, 3H, CH_3_), 4.00 (q, 2H, *J =* 7.2 Hz, CH_2_), 7.67 (d, 2H, *J =* 7.8 Hz, ArH’s), 7.89 (d, 2H, *J =* 7.8 Hz, ArH’s); ^13^C NMR (75.46 MHz, DMSO-d_6_) δ: 13.56, 28.88, 59.58, 116.48, 121.33, 123.58, 125.47, 126.05, 127.89, 132.11, 141.05, 146.36, 159.87, 192.01. MS (m/z): 394 (M^+^). (Calc.: C, 48.74; H, 3.07; N, 7.11. C_16_H_12_F_6_N_2_O_3_ Found: C, 49.05; H, 2.94; N,6.93).

#### Ethyl 4-benzoyl-1-(4-trifluoromethylphenyl)-5-(trifluoromethyl)-1H-pyrazole-3-carboxylate (11j)

M.p. = 207–208°C; IR (KBr): 1722, 1701 (2 C = O), 1602 (C = N) cm^-1^; ^1^H NMR (300 MHz, DMSO-d_6_) δ: 1.16 (t, 3H, *J =* 7.2 Hz CH_3_), 3.91(q, 2H, *J =* 7.2 Hz, CH_2_), 7.51-7.88 (m, 9H, ArH’s); ^13^C NMR (75.46 MHz, DMSO-d_6_) δ: 13.98, 59.04, 114.52, 118.12, 121.54, 121.55, 125.87, 126.02, 128.11, 128.95, 132.05, 132.97, 134.23, 142.31, 148.00, 162.45, 196.10. MS (m/z): 456 (M^+^). (Calc.: C, 55.27; H, 3.09; N, 6.14. C_21_H_14_F_6_N_2_O_3_ Found: C, 55.58; H, 2.95; N, 5.97).

#### Ethyl 4-acetyl-1-(4-chlorophenyl)-5-(trifluoromethyl)-1H-pyrazole-3-carboxylate (11k)

M.p. = 227–229°C; IR (KBr): 1725, 1705 (2 C = O), 1602 (C = N) cm^-1^; ^1^H NMR (300 MHz, DMSO-d_6_) δ: 1.19 (t, 3H, *J =* 7.00 Hz, CH_3_), 2.21 (s, 3H, CH_3_), 3.95 (q, 2H, *J =* 7.00 Hz, CH_2_), 7.60 (d, 2H, *J =* 7.8 Hz, ArH’s), 7.52 (d, 2H, *J =* 7.8 Hz, ArH’s); ^13^C NMR (75.46 MHz, DMSO-d_6_) δ: 13.52, 28.06, 58.56, 115.18, 121.33, 125.18, 127.51, 132.55, 133.00, 146.15, 159.11, 190.00. MS (m/z): 360 (M^+^). (Calc.: C, 49.95; H, 3.35; N, 7.77. C_15_H_12_ClF_3_N_2_O_3_ Found: C, 50.21; H, 3.27; N,7.59).

#### Ethyl 4-benzoyl-1-(4-chlorophenyl)-5-(trifluoromethyl)-1H-pyrazole-3-carboxylate (11 l)

M.p. = 250–251°C; IR (KBr): 1723, 1703 (2 C = O), 1602 (C = N) cm^-1^; ^1^H NMR (300 MHz, DMSO-d_6_) δ: 0.95 (t, 3H, *J =* 7.2 Hz CH_3_), 3.99(q, 2H, *J =* 7.2 Hz, CH_2_), 7.62-7.95 (m, 9H, ArH’s); ^13^C NMR (75.46 MHz, DMSO-d_6_) δ: 13.90, 59.22, 114.00, 117.45, 121.17, 126.89, 128.12, 128.57, 132.00, 133.09, 133.89, 139.11, 147.52, 159.47, 192.13. MS (m/z): 422 (M^+^). (Calc.: C, 56.82; H, 3.34; N, 6.63. C_20_H_14_ClF_3_N_2_O_3_ Found: C, 57.03; H, 3.28; N, 6.48).

## Competing interests

The authors declare that they have no competing interests.

## Authors’ contributions

The current study is the outcome of constructive discussions between ASA and TSS, ASA and TSS carry out the synthesis and characterization experiments. HMA carried out the GC-MS analysis and ASA and TSS carried out the ^1^H NMR, ^13^C NMR and 2D NMR. ASA and TSS carried out the elemental analyses. ASA and TSS, were involved in revising the manuscript. All authors have read and approve of the final manuscript.

## References

[B1] Elguero J Katrizky AR, Rees CW, Scriven EFV Comprehensive Heterocyclic Chemistry II, Volume 3 1996 Oxford: Pergamon 1

[B2] Katzenellenbogen BS Katzenellenbogen JA Pyrazole ligands: structure−affinity/activity relationships and estrogen receptor-α-selective agonists J Med Chem 2000 43 4934 4947 10.1021/jm000170m 11150164

[B3] Fink BE Mortensen DS Stauffer SR Aron ZD Katzenellenbogen JA Novel structural templates for estrogen-receptor ligands and prospects for combinatorial synthesis of estrogens Chem Biol 1999 6 205 221 10.1016/S1074-5521(99)80037-4 10099132

[B4] Kraichely D Sun J Katzenellenbogen J Katzenellenbogen B Conformational changes and coactivator recruitment by Novel Ligands for estrogen receptor-α and estrogen receptor-β: correlations with biological character and distinct differences among SRC coactivator family members Endocrinology 2000 141 3534 3545 10.1210/en.141.10.3534 11014206

[B5] Baraldi PG Tabrizi MA Romagnoli R Fruttarolo F Merighi S Varani K Gessi S Borea PA pyrazolo[4,3-e]1,2,4-triazolo[1,5-c]pyrimidine ligands, new tools to characterize A_3_ adenosine receptors in human tumor cell lines Curr Med Chem 2005 12 1319 1329 10.2174/0929867054020963 15974999

[B6] Stauffer SR Huang YR Aron ZD Coletta CJ Sun J Katzenellenbogen BS Katzenellenbogen JA Triarylpyrazoles with basic side chains: development of pyrazole-based estrogen receptor antagonists Bioorg Med Chem 2001 9 151 161 10.1016/S0968-0896(00)00226-1 11197335

[B7] Ashton WT Hutchins SM Greenlee WJ Doss GA Chang RSL Lotti VJ Faust KA Chen TB Zingaro GJ Kivlighn SD Siegl PKS Nonpeptide angiotensin II antagonists derived from 1H-pyrazole-5-carboxylates and 4-aryl-1H-imidazole-5-carboxylates J Med Chem 1993 36 3595 3605 10.1021/jm00075a014 8246227

[B8] Flynn DL Belliotti TR Boctor AM Conor DT Kostlan CR Nies DE Ortwine DF Schrier DJ Sircar JC Styrylpyrazoles, styrylisoxazoles, and styrylisothiazoles. Novel 5-lipoxygenase and cyclooxygenase inhibitors J Med Chem 1991 34 518 525 10.1021/jm00106a006 1847426

[B9] Ma JA Cahard D Asymmetric fluorination, trifluoromethylation, and perfluoroalkylation reactions Chem Rev 2004 104 6119 6146 10.1021/cr030143e 15584697

[B10] Pavlik JW Ayudhya TIN Tantaynon S Condensation of methyl-hydrazine with 4-ethoxy-1,1,1-trifluoro-3-buten-2-one. a reinvestigation J Het Chem 2002 39 1025 1027 10.1002/jhet.5570390526

[B11] Pavlik JW Ayudhya TIN Tantaynon S Synthesis of some trifluoromethyl substituted 1-methylpyrazoles J Het Chem 2003 40 1087 1089 10.1002/jhet.5570400619

[B12] Singh SP Kumar D A facile synthesis of 5-methyl-1-(phenyl/heterocyclyl)-4-trifluoroacetylpyrazoles J Chem Res 1997 142 143 10.1039/A606444B

[B13] Nenajdenko VG Reznichenko AL Balenkova ES Regioselective synthesis of 1-aryl-4-bromo-5-trifluoromethylpyrazoles Russ Chem Bull 2006 55 172 10.1007/s11172-006-0233-z

[B14] Nenajdenko VG Sanin AV Balenkova ES (a) Preparation of α,β-unsaturated ketones bearing a trifluoromethyl group and their application in organic synthesis Molecules 1997 2 186 232 (b) Nenajdenko, VG; Sanin, AV; Balenkova, ES, C-Alkenylation of pyrimidine nucleosides and their analogues*, Russ Chem Rev,* **1999**, *68*, 483–504. (c) Druzhinin, SV; Balenkova, ES; Nenajdenko, VG. Recent advances in the chemistry of α,β-unsaturated trifluoromethylketones, *Tetrahedron,* **2007**, *63*, 7753–7808. (d) Erian, AW; Recent trends in the Chemistry of fluorinated five and six-membered heterocycles, *J Het Chem* **2001**, *38*, 793–808. (e) Kumar, V; Aggarwal, R; Singh, SP; Reaction of hydrazines and hydroxylamine with trifluoromethyl-β-diketones: synthesis of trifluoromethylpyrazole and isoxazole derivatives, *Heterocycles,* **2008**, *75*, 2893–2929 10.3390/21200186

[B15] Oh LM Synthesis of celecoxib via 1,3-dipolar cycloaddition Tetrahedron Lett 2006 47 7943 7946 10.1016/j.tetlet.2006.08.138

[B16] Jenkins CL Kochi JK Solvolytic routes via alkylcopper intermediates in the electron-transfer oxidation of alkyl radicals J Am Chem Soc 1972 94 843 855 10.1021/ja00758a024

[B17] Kobayashi Y Taguchi T Tokuno E Synthesis of 1,4-diketones by oxidative coupling of kitone enolates and trimethylsilyl enol ethers with cupric trifluoromethanesulfonate Tetrahedron Lett 1977 18 3741 3742 10.1016/S0040-4039(01)83341-4

[B18] Andrist AH Angello RM Wolfe DC Facile preparation of optically active c-2, t-3-dimethyl-r-1-methoxycyclopropane J Org Chem 1978 43 3422 3423 10.1021/jo00411a047

[B19] Hubert AJ Feron A Warin R Teyssie P Synthesis of iminoaziridines from carbodiimides and diazoesters: a new example of transition metal salt catalysed reactions of carbenes Tetrahedron Lett 1976 17 1317 1318 10.1016/S0040-4039(00)78050-6

[B20] Nagayoshi K Sato T One-step synthesis of oxazoles from ketones and nitriles using copper(II) trifluoromethanesulfonate as a key reagent Chem Lett 1983 1355 1356

[B21] Yadav JS Reddy BVS Baishya G Narsaiah AV Copper(II) triflate immobilized in [bmim]BF_4_ ionic liquid: an efficient reaction medium for michael addition of β-ketoesters to acceptor-activated alkenes Chem Lett 2005 34 102 103 10.1246/cl.2005.102

[B22] Yadav JS Reddy BVS Satheesh G InBr_3_/Cu(OTf)_2_-catalyzed C-alkylation of pyrroles and indoles with α-diazocarbonyl compounds Tetrahedron Lett 2003 44 8331 8334 10.1016/j.tetlet.2003.09.031

[B23] Paraskar AS Dewkar GK Sudalai A Cu(OTf)_2_: a reusable catalyst for high-yield synthesis of 3,4-dihydropyrimidin-2(1H)-ones Tetrahedron Lett 2003 44 3305 3308 10.1016/S0040-4039(03)00619-1

[B24] Kobayashi S Sugiura M Kitagawa H Lam WW-L Rare-earth metal triflates in organic synthesis Chem Rev 2002 102 2227 2302 10.1021/cr010289i 12059268

[B25] Cravotto G Cintas P Power ultrasound in organic synthesis: moving cavitational chemistry from academia to innovative and large-scale applications Chem Soc Rev 2006 35 180 196 10.1039/b503848k 16444299

[B26] Bretanha LC Teixeira VE Ritter M Siqueira GM Cunico W Pereira CMP Freitag RA Ultrasound-promoted synthesis of 3-trichloromethyl-5-alkyl(aryl)-1,2,4-oxadiazoles Ultrason Sonochem 2011 18 704 707 10.1016/j.ultsonch.2010.09.016 21115383

[B27] Duarte A Cunico W Pereira CMP Flores AFC Freitag RA Siqueira GM Ultrasound promoted synthesis of thioesters from 2-mercaptobenzoxa(thia)zoles Ultrason Sonochem 2010 17 281 283 10.1016/j.ultsonch.2009.08.004 19740694

[B28] Machado P Lima GR Rotta M Bonacorso HG Zanatta N Martins MAP Efficient and highly regioselective synthesis of ethyl 1-(2,4-dichlorophenyl)-1H-pyrazole-3-carboxylates under ultrasound irradiation Ultrason Sonochem 2011 18 293 299 10.1016/j.ultsonch.2010.06.009 20638886

[B29] Pizzuti L Martins PLG Ribeiro BA Quina FH Pinto E Flores AFC Venzke D Pereira CMP Efficient sonochemical synthesis of novel 3,5-diaryl-4,5-dihydro-1H-pyrazole-1-carboximidamides Ultrason Sonochem 2010 17 34 37 10.1016/j.ultsonch.2009.06.013 19632139

[B30] Venzke D Flores AFC Quina FH Pizzuti L Pereira CMP Rapid decolorization of azo dyes in aqueous solution by an ultrasound-assisted electrocatalytic oxidation process Ultrason Sonochem 2010 17 370 374 10.1016/j.ultsonch.2009.10.002 19879175

[B31] Tiwari V Parvez A Meshram J Benign methodology and improved synthesis of 5-(2-chloroquinolin-3-yl)-3-phenyl-4,5-dihydroisoxazoline using acetic acid aqueous solution under ultrasound irradiation Ultrason Sonochem 2011 18 911 916 10.1016/j.ultsonch.2010.12.003 21227731

[B32] Sant’Anna GS Machado P Sauzem PD Rosa FA Rubin MA Ferreira J Bonacorso HG Zanatta N Martins MAP Ultrasound promoted synthesis of 2-imidazolines in water: a greener approach toward monoamine oxidase inhibitors Bioorg Med Chem Lett 2009 19 546 549 10.1016/j.bmcl.2008.03.001 19064321

[B33] Mamaghani M Loghmanifar A Taati MR An efficient one-pot synthesis of new 2-imino-1,3-thiazolidin-4-ones under ultrasonic conditions Ultrason Sonochem 2011 18 45 48 10.1016/j.ultsonch.2010.05.009 20579925

[B34] Pereira CMP Stefani HA Guzen KP Orfao ATG Improved synthesis of benzotriazoles and 1-acylbenzotriazoles by ultrasound irradiation Lett Org Chem 2007 4 43 46

[B35] Stefani HA Oliveira CB Almeida RB Pereira CMP Braga RC Cella R Borges VC Savegnago L Nogueira CW Dihydropyrimidin-(2H)-ones obtained by ultrasound irradiation: a new class of potential antioxidant agents Eur J Med Chem 2006 41 513 518 10.1016/j.ejmech.2006.01.007 16516351

[B36] Rodrigues-Santos C Echevarria A Convenient syntheses of pyrazolo[3,4-b]pyridin-6-ones using either microwave or ultrasound irradiation Tetrahedron Lett 2011 52 336 346 10.1016/j.tetlet.2010.11.054

[B37] Abd El-Rahman NM Saleh TS Mady MF Ultrasound assisted synthesis of some new 1,3,4-thiadiazole and bi(1,3,4-thiadiazole) derivatives incorporating pyrazolone moiety Ultrason Sonochem 2009 16 70 74 10.1016/j.ultsonch.2008.05.001 18567527

[B38] Saleh TS Abd-El-Rahman NM Ultrasound promoted synthesis of substituted pyrazoles and isoxazoles containing sulphone moiety Ultrason Sonochem 2009 16 237 242 10.1016/j.ultsonch.2008.07.012 18835210

[B39] Saleh TS Abd El-Rahman NM Elkateb AA Shaker NO Mahmoud NA Gabal SA Ultrasound promoted synthesis of some novel fused pyrans Ultrason Sonochem 2012 19 491 497 10.1016/j.ultsonch.2011.10.008 22119428

[B40] Ahmed NS Saleh TS El-Mossalamy EH An Efficiently Sonochemical Synthesis Of novel pyrazoles, bipyrazoles and pyrazol-3-ylPyrazolo[3,4-d]pyrimidines incorporating 1H-benzoimidazole Curr Org Chem 2013 17 194 202 10.2174/1385272811317020016

[B41] Mokhtar M Saleh TS Ahmed NS Al-Thabaiti SA Al-Shareef RA An eco-friendly N-sulfonylation of amines using stable and reusable Zn–Al–hydrotalcite solid base catalyst under ultrasound irradiation Ultrason Sonochem 2011 18 172 176 10.1016/j.ultsonch.2010.05.001 20627793

[B42] Saleh TS Eldebss TMA Albishri HM Ultrasound assisted one-pot, three-components synthesis of pyrimido[1,2-a]benzimidazoles and pyrazolo[3,4-b]pyridines: a new access via phenylsulfone synthon Ultrason Sonochem 2012 19 49 55 10.1016/j.ultsonch.2011.05.003 21723178

[B43] Shaaban MR Saleh TS Osman FH Farag AM Regioselective synthesis of some novel pyrazoles, isoxazoles, pyrazolo[3,4-*d*]pyridazines and isoxazolo[3,4-*d*]pyridazines pendant to benzimidazole J Heterocycl Chem 2007 44 177 181 10.1002/jhet.5570440127

[B44] Shaaban MR Saleh TS Farag AM Synthesis and antimicrobial evaluation of new thiophene and 1,3,4-thiadiazole derivatives Heterocycles 2009 78 151 159 10.3987/COM-08-11512

[B45] Shaaban MR Saleh TS Farag AM An efficient single step synthesis of pyridazine, pyrazolo[5,1-*c*]-1,2,4-triazine, 1,2,4-triazolo[5,1-*c*]-1,2,4-triazine and 1,2,4-triazino[4,3-*a*]benzimidazole derivatives Heterocycles 2009 78 699 706 10.3987/COM-08-11559

[B46] Abdel-Aziz HA Saleh TS El-Zahabi HSA Facile synthesis and in-vitro antitumor activity of some pyrazolo[3,4-b]pyridines and pyrazolo[1,5-a]pyrimidines linked to a thiazolo[3,2-a]benzimidazole moiety Arch Pharm 2010 343 24 30 10.1002/ardp.200900082 19921685

[B47] Mokhtar M Saleh TS Basahel SN Mg–Al hydrotalcites as efficient catalysts for aza-Michael addition reaction: a green protocol Journal of Molecular Catalysis A: Chemical 2012 353–354 122 131

[B48] Al-Bogami AS Synthesis and Characterization of Some New N-(Substituted 4-Methylene-2-oxo-4*H*-benzo[e][1,3]oxazin-3-yl)isonicotinamide Asian J Chem 2011 23 3045 3049

[B49] Al-Bogami AS Almajid AM Al-Saad MA Mosa AM Almazroa SA Alkhathlan HZ Cyclization of Hydrazones of 2-Acetyl-1-naphthol and 1-Acetyl-2-naphthol with Triphosgene. Synthesis of Spiro Naphthoxazine Dimers Molecules 2009 14 2147 2159 10.3390/molecules14062147 19553888 PMC6254203

[B50] Al-Bogami AS Synthesis of 4-methylene-1,3-naphthoxazines by the reaction of imines with triphosgene Synth Commun 2011 41 2952 2958 10.1080/00397911.2010.515767

[B51] Al-Bogami AS Karama U Mousa AA Khan V Al-mazroa SA Alkhathlan HZ Simple and efficient one step synthesis of functionalized flavanones and chalcones Orient J Chem 2012 28 619 626 10.13005/ojc/280201

[B52] Park JD Brown HA Lacher JR A study of some fluorine-containing β-diketones J Am Chem Soc 1953 75 4753 4756 10.1021/ja01115a041

[B53] Niwa J Yamazaki M Takeuchi T A carbon-13 nmr study of the direction of enolization of trifluoroacetylacetone Chem Lett 1975 707 710

[B54] Yamaguchi Y Katsuyama I Funabiki K Matsu M Shibata K A new expedient route to 2,6-diaryl-3-cyano-4-(trifluoromethyl)pyridines J Het Chem 1998 35 805 810 10.1002/jhet.5570350405

[B55] Loupy A Luche J-L Luche J-L Sonochemistry in biphasic system Synthetic Organic Sonochemistry 1998 New York: Plenum press 107 165

[B56] Suslick KS Sonochemistry Science 1990 247 1439 1445 10.1126/science.247.4949.1439 17791211

[B57] Farag AM Algharib MS Synthesis and reactions of C-(2-thenoyl)-N-arylformhydrazidoyl bromides Org Prep Proced Int 1988 20 521 526 10.1080/00304948809356298

[B58] Iwata S Moroi Y Mitsuhashi K Tanaka K Nippon Kagaku Kaishi, 1992, 1144 Chem Abstr 1993 118 59627

